# Measurement of skeletal muscle glycogen status in critically ill patients: a new approach in critical care monitoring

**DOI:** 10.1186/cc14480

**Published:** 2015-03-16

**Authors:** I San Millan, J Hill, P Wischmeyer

**Affiliations:** 1 University of Colorado, Aurora, CO, USA

## Introduction

Critically ill patients experience hypermetabolism increas ing substrate utilization, especially glucose oxidation. Glycogen is the main source of glucose in the body, being 85% and 15% stored in skeletal muscle and liver respectively. Since glycogen stores are limited we evaluated the hypothesis that critical illness could be associated with glycogen depletion leading to skeletal muscle catabolism for gluconeogenesis and eventually resulting in cachexia, an important determinant of future ICU survival and ICU-acquired weakness.

## Methods

Nine critically ill patients (58.75 ± 25 to 75 years old) with an ICU stay from 1 day to 5 weeks were evaluated for skeletal muscle glycogen content using a rapid, non-invasive high-frequency ultrasound methodology (MuscleSound®, Denver, CO, USA). Scans were obtained from the rectus femoris and vastus lateralis muscles. Glycogen content was measured with a score from 0 to 100 according to the MuscleSound® scale. Patients had a variety of primary diagnoses including septic shock (*n *= 3), hemorrhagic shock/abdominal hypertension (*n *= 1), hypovolemic shock/post major oncologic surgery (*n *= 1), trauma (*n *= 3), and burn injury (*n *= 1).

## Results

Six out of nine patients had no glycogen present in the muscle (score = 0). The other three patients had glycogen scores between 5 and 15 which are well below scores of healthy individuals (reference 50 to 70). As a comparison we collected post-competition levels in competitive athletes, which decrease their glycogen stores (score 15 to 25) but are well above those of most critically ill patients we have studied.

## Conclusion

This is the first time that muscle glycogen stores have been evaluated in critical illness. Our data show severe glycogen depletion in ICU patients which probably leads to muscle catabolism necessary for gluconeogenesis, eventually resulting in cachexia. This finding poses severe metabolic challenges for ICU patients in which interfering with recovery can contribute to poor survival. In light of our findings, re-evaluation of nutritional protocols and potential anabolic/anticatabolic therapy to decrease muscle catabolism may improve survival. Different therapeutics that may prevent hypermetabolism (such as beta-blockers) should be re-evaluated along with anabolic agents (that is, oxandrolone) which could counteract the severe catabolic response in critical illness. Monitoring of muscle glycogen repletion could signal the transition from the catabolic to anabolic phase.

